# Biogeographic study of human gut-associated crAssphage suggests impacts from industrialization and recent expansion

**DOI:** 10.1371/journal.pone.0226930

**Published:** 2020-01-15

**Authors:** Tanvi P. Honap, Krithivasan Sankaranarayanan, Stephanie L. Schnorr, Andrew T. Ozga, Christina Warinner, Cecil M. Lewis

**Affiliations:** 1 Laboratories of Molecular Anthropology and Microbiome Research, University of Oklahoma, Norman, Oklahoma, United States of America; 2 Department of Anthropology, University of Oklahoma, Norman, Oklahoma, United States of America; 3 Department of Microbiology and Plant Biology, University of Oklahoma, Norman, Oklahoma, United States of America; Leibniz-Institute DSMZ, GERMANY

## Abstract

CrAssphage (cross-assembly phage) is a bacteriophage that was first discovered in human gut metagenomic data. CrAssphage belongs to a diverse family of crAss-like bacteriophages thought to infect gut commensal bacteria belonging to *Bacteroides* species. However, not much is known about the biogeography of crAssphage and whether certain strains are associated with specific human populations. In this study, we screened publicly available human gut metagenomic data from 3,341 samples for the presence of crAssphage *sensu stricto* (NC_024711.1). We found that crAssphage prevalence is low in traditional, hunter-gatherer populations, such as the Hadza from Tanzania and Matses from Peru, as compared to industrialized, urban populations. Statistical comparisons showed no association of crAssphage prevalence with variables such as age, sex, body mass index, and health status of individuals. Phylogenetic analyses show that crAssphage strains reconstructed from the same individual over multiple time-points, cluster together. CrAssphage strains from individuals from the same study population do not always cluster together. Some evidence of clustering is seen at the level of broadly defined geographic regions, however, the relative positions of these clusters within the crAssphage phylogeny are not well-supported. We hypothesize that this lack of strong biogeographic structuring is suggestive of an expansion event within crAssphage. Using a Bayesian dating approach, we estimate that this expansion has occurred fairly recently. Overall, we determine that crAssphage presence is associated with an industrialized lifestyle and the absence of strong biogeographic structuring within global crAssphage strains is likely due to a recent population expansion within this bacteriophage.

## Introduction

The virome harbors the most abundant and diverse set of genes on earth, with deep impacts on host biology, including the very architecture of host genomes [1]. Often described as the “dark matter” of biology [2], the virome has an underexplored role in human health [3]. Much of the human gut virome is composed of double-stranded DNA bacteriophages [4], which are argued to be the primary regulators of bacterial biomass [5]. Bacteriophages are hypothesized to impact the human gut [4]; for example, through potentially transmitting bacterial antibiotic resistance genes and facilitating bacterial carbohydrate utilization [6]. The majority of the bacteriophages found in the human gut belong to the families *Microviridae*, *Siphoviridae*, *Podoviridae*, and *Myoviridae* [7]; however, our knowledge of the true bacteriophage diversity is limited [8]. While the human virome is a component of the human microbiome, it is not as amenable to high throughput taxonomic characterization as the archaeal and bacterial subsets. Specifically, viruses lack “universal” taxonomically diagnostic marker genes (for example, the 16S ribosomal RNA gene in archaea and bacteria), preventing characterization using current amplicon-based sequencing strategies.

Recent advances in metagenomic sequencing and development of data mining tools now allow for large-scale studies of human gut bacteriophage diversity using shotgun-sequencing data from human fecal samples [8]. From such data, one particular DNA bacteriophage, crAssphage, appears to be remarkably abundant in the human gut [9]. CrAssphage (cross-assembly phage) was named after the crAss (cross-assembly) software originally used to discover the bacteriophage [9]. CrAssphage is prevalent in available gut metagenomic data from the U.S. [10], comprising up to 90% of viral particle-derived metagenomes and up to 22% of reads in a total fecal community from the U.S. National Institutes of Health’s Human Microbiome Project cohort [11]. Moreover, crAssphage is up to six times more abundant than all other known bacteriophages that could be reconstructed from these publicly available metagenomes [9]. Bacteria belonging to phylum Bacteroidetes, which are commonly found gut commensals, were predicted to be the host of this bacteriophage [9].

A recent study has shown that crAssphage, which is now referred to as crAssphage *sensu stricto*, is actually a member of a diverse group of crAss-like bacteriophages [12]. The first isolated member of this group, ΦCrAss001, shows podovirus-like morphology and infects the bacterium, *Bacteroides intestinalis* [13]. In addition to the human gut, crAss-like phages are found in nonhuman primate and termite guts, terrestrial and groundwater sources, and oceanic environments [12, 14–16]. Across metagenomic studies, crAssphage *sensu stricto* is nearly exclusively associated with the human gut microbiome [9, 12] and has been proposed as a potential biomarker for fecal contamination [16–20].

Recent research suggests that crAssphage may be present in infants as early as one-month after birth [21], which is partly explained by the fact that *Bacteroides* species are among the most abundant members of the gut microbiomes of newborns [22]. It has also been suggested that while most adults contain a single dominant crAssphage strain [21], a minority can carry multiple (hundreds) crAssphage strains [16]. However, the means by which crAssphage is initially acquired [14, 21], strain variation within an individual [16], overall geographic distribution [16, 23], and prevalence in other environmental reservoirs has not been fully elucidated. Although crAssphage has been detected globally using PCR-based assays [14, 16], a systematic analysis of the strain biogeography of crAssphage is necessary, considering the diverse range of currently reported crAssphage strains and the existence of other crAss-like phages in the human gut [12] that may confound crAssphage detection using PCR-based assays.

Here, we report strain-level prevalence and diversity of crAssphage *sensu stricto* observed across 3,341 gut metagenomic samples originating from globally distributed human populations (Table 1). In this study, we consider genetically non-identical crAssphage sequences as different crAssphage strains [24]. Further, we evaluate crAssphage prevalence, abundance, and strain diversity as a function of biogeography, human dietary lifestyle and variables including age, sex, body mass index (BMI), and health status.

**Table 1:** Publicly available gut metagenomic datasets used in this study.

Dataset	Description	Samples	Individuals	Accession	Reference
BCK	Urban mother-infant pairs from Sweden	400	200	ERP005989	[22]
CNA	Urban individuals of Cheyenne, Arapaho, and non-native ancestry from Oklahoma, USA	61	61	PRJNA299502	[25]
HAD	Hadza hunter-gatherers from Tanzania	67	67	SRP056480, SRP110665	[26], [27]
HMP	Urban individuals from USA	204	123	phs000228.v3.p1	[11]
ITA	Urban individuals from Italy	11	11	SRP056480	[26]
ISR	Urban individuals from Israel	950	851	PRJEB11532	[28]
KRL	Urban individuals from Sweden	145	145	ERP002469	[29]
LIU	Traditional pastoralists from Mongolia	110	110	SRP080787	[30]
MAT	Matses hunter-gatherers from Peru	25	25	PRJNA268964	[31]
MHC	Urban individuals from China	363	363	SRA045646, SRA050230	[32]
MHE	Urban individuals from Sweden and Denmark	756	606	ERP003612, ERP004605, ERP002061	[33], [34], [35]
XIE	Urban twin-pairs from the UK	249	249	ERP010708	[36]

## Results and discussion

### CrAssphage screening

Publicly available gut metagenome data from 3,341 samples were screened for the presence of crAssphage *sensu stricto* using a two-pronged approach: first, a direct reference-based mapping to crAssphage (NC_024711.1) using Bowtie2 [37], and second, a *de novo* assembly using MEGAHIT [38] followed by a protein-protein BLAST search (BLASTP) [39] against the crAssphage proteins (see Methods). Mapping and assembly statistics are provided in S1 Table.

Using the reference-based mapping approach, we identified 614 samples wherein a high-quality crAssphage strain could be reconstructed (>70% of the genome at >10-fold mean coverage). However, this approach is suitable only for samples containing a single dominant crAssphage strain. 60 samples showed more than 100 heterozygous sites (which corresponds to 0.1% of the total genome), with the maximum being 1,118 heterozygous sites in a single sample. In order to determine whether the samples comprised multiple crAss-like phages, we performed a *de novo* assembly of each metagenome. We used BLASTP to query the predicted open reading frames (ORFs) for each sample against the crAssphage reference (NC_024711.1) proteins. The BLASTP hits were then filtered to include only those which showed 95% query coverage and 95% identity to crAssphage proteins. These criteria were chosen to avoid false positive hits from crAss-like phages, since the average pairwise identity between members of the crAss-like phage family ranges from 20–40% [12, 40]. Using this approach, we identified 963 samples wherein at least one crAssphage protein was recovered (S2 Table). We were not able to recover all 90 proteins from a single sample, with the maximum number of crAssphage proteins recovered from a single sample being 67. We hypothesize that this is due to the limitations of our *de novo* metagenomic assembly as well as the stringency of our BLASTP search parameters, wherein our approach is likely biased towards avoiding false positive hits. However, our approach is not biased towards recovery of specific crAssphage proteins, since all 90 crAssphage reference proteins were recovered across all samples. Since we were only able to recover a maximum of 67 proteins for a single sample, we decided to use a 50% coverage threshold to call a sample “crAss-positive”, i.e. at least 33 crAssphage proteins had to be recovered from the sample. Using this criterion, 719 samples were found to be crAss-positive.

### crAssphage prevalence

To assess the prevalence of crAssphage among individuals from each study population as well as potential associations with health, individuals were divided into categories based on health status (Table 2). For some datasets (BCK, HMP, ISR, and MHE), multiple samples were available from the same individual; in this case, an individual was considered crAss-positive if at least one sample from that individual was positive. None of the HAD and ITA samples, acquired from Hadza individuals from Tanzania (N = 67) and urban Italians (N = 11), respectively, were crAss-positive. For the MAT samples acquired from Matses individuals from Peru (N = 25), only two were crAss-positive. The Hadza peoples from Tanzania as well as the Matses from the Amazon jungle of Peru have a subsistence practice primarily based on hunting and gathering [31, 41]. Among healthy, urban individuals leading an industrialized lifestyle, crAssphage prevalence ranged from 14.0% among Chinese individuals from the MetaHIT cohort (MHC; N = 185) to 35.7% among U.S. residents from the HMP cohort (N = 123). CrAssphage prevalence showed no significant associations with human health status (Table 2).

**Table 2:** Association of health status with prevalence of crAssphage.

Dataset	Total number of individuals	Health Status						
		Healthy	IGT	T2D	CD	IBD	UC	Not specified
**BCK**	**200**	39 (200)	0 (0)	0 (0)	0 (0)	0 (0)	0 (0)	0 (0)
**CNA**	**61**	14 (37)	0 (0)	11 (18)	0 (0)	0 (0)	0 (0)	2 (6)
**HAD**	**67**	0 (0)	0 (0)	0 (0)	0 (0)	0 (0)	0 (0)	0 (67)
**HMP**	**123**	47 (123)	0 (0)	0 (0)	0 (0)	0 (0)	0 (0)	0 (0)
**ISR**	**851**	185 (851)	0 (0)	0 (0)	0 (0)	0 (0)	0 (0)	0 (0)
**ITA**	**11**	0 (11)	0 (0)	0 (0)	0 (0)	0 (0)	0 (0)	0 (0)
**KRL**	**145**	15 (43)	12 (49)	20 (53)	0 (0)	0 (0)	0 (0)	0 (0)
**LIU**	**110**	11 (110)	0 (0)	0 (0)	0 (0)	0 (0)	0 (0)	0 (0)
**MAT**	**25**	2 (25)	0 (0)	0 (0)	0 (0)	0 (0)	0 (0)	0 (0)
**MHC**	**363**	37 (185)	0 (0)	23 (176)	0 (0)	0 (0)	0 (0)	1 (2)
**MHE**	**606**	87 (350)	0 (0)	0 (0)	2 (9)	7 (25)	12 (48)	53 (174)
**XIE**	**249**	64 (211)	0 (0)	5 (10)	0 (0)	0 (0)	0 (0)	12 (28)

Values indicate number of crAss-positive individuals (total number of individuals in the category). Health status categories refer to: IGT–Impaired Glucose Tolerance, T2D –Type 2 Diabetes, CD–Crohn’s Disease, IBD–Inflammatory Bowel Disorder, UC–Ulcerative Colitis.

Our findings suggest that crAssphage prevalence may be associated with an industrialized lifestyle. A similar finding was reported in a manuscript that was published while our manuscript was under review [16]. This latter study found that gut metagenomic data for rural Malawi and Amazonas individuals from Venezuela [42] as well as the ~5300-year old Tyrolean Iceman [43] show very low numbers of crAssphage sequences. The association of crAssphage with industrialized practices can also be partially explained by its putative bacterial host, *Bacteroides* species [9]. CrAss-like phages can infect *Bacteroides* [13], although it is not known if they are limited to this host genus [44]. Gut microbiome studies have shown that *Bacteroides* tend to be more abundant in industrialized human populations as compared to traditional peoples [45], and the Hadza and Matses individuals are consistent with this pattern [31, 41]. However, the two crAss-positive Matses individuals do not show higher relative abundance of *Bacteroides* as compared to crAss-negative Matses individuals (S1 Fig).

For healthy individuals, we also analyzed metadata, such as age, sex, and BMI, to study potential associations with prevalence of crAssphage. The HAD, ISR, ITA, LIU, and MAT datasets were excluded from this analysis due to unavailability of metadata or insufficient number of crAss-positive individuals. There were no statistically significant differences in the prevalence of crAssphage among individuals based on age, sex, or BMI (Table 3).

**Table 3:** Association of age, sex, and BMI of individuals with prevalence of crAssphage.

Dataset	CNA	HMP	KRL	MHC	MHE	XIE
**Age**						
**18–40**	9 (23)	47 (123)	0 (0)	16 (74)	11 (35)	1 (4)
**41–65**	4 (12)	0 (0)	0 (0)	19 (103)	47 (215)	44 (134)
**> 65**	1 (2)	0 (0)	15 (43)	2 (7)	2 (19)	19 (73)
**Sex**						
**Male**	5 (18)	30 (65)	0 (0)	19 (95)	31 (128)	0 (0)
**Female**	9 (19)	17 (58)	15 (43)	18 (90)	29 (142)	64 (211)
**BMI**						
**< 18.5**	0 (0)	0 (0)	1 (1)	2 (17)	0 (3)	0 (4)
**18.5–24.9**	2 (13)	29 (68)	6 (18)	17 (104)	29 (130)	35 (108)
**> = 25.0**	12 (24)	18 (55)	8 (24)	18 (64)	58 (207)	29 (98)

Values denote number of crAss-positive individuals (total number of individuals in the category)

### Phylogenetic analyses

To study the phylogenetic relationships between the crAssphage strains, we selected ten genes which are present in all members of the crAss-like phage family [12]. These genes include those encoding the five putative capsid proteins, a single-stranded DNA-binding protein, a DNA-G family primase, a PD-(D/E)XK family nuclease, and two hypothetical proteins. We then identified a subset of 232 samples wherein only one sample ORF matched these crAssphage reference proteins, suggesting the presence of a single strain of crAssphage in the samples. Furthermore, we confirmed that these ORFs had similar depths of coverage to verify that they represent the same viral genome. We also included the reference crAssphage genome (NC_024711.1), resulting in a total of 233 taxa. A nucleotide alignment comprising these ten genes contained 12,642 sites. A Maximum Likelihood (ML) tree of the 233 strains based on this multi-gene alignment is given in S2 Fig.

According to the ML tree, crAssphage strains from the same individual cluster together. While strains from individuals belonging to the same population/study dataset do cluster together, many such crAssphage clusters are found in each population. Unfortunately, the relative positions of these clusters could not be well-established due to insufficient bootstrap support. Interestingly, we found that the strain from a crAss-positive Matses individual clusters with crAssphage strains from European individuals from the MetaHIT cohort, suggesting that it belongs to a widespread crAssphage clade.

**Fig 1:**
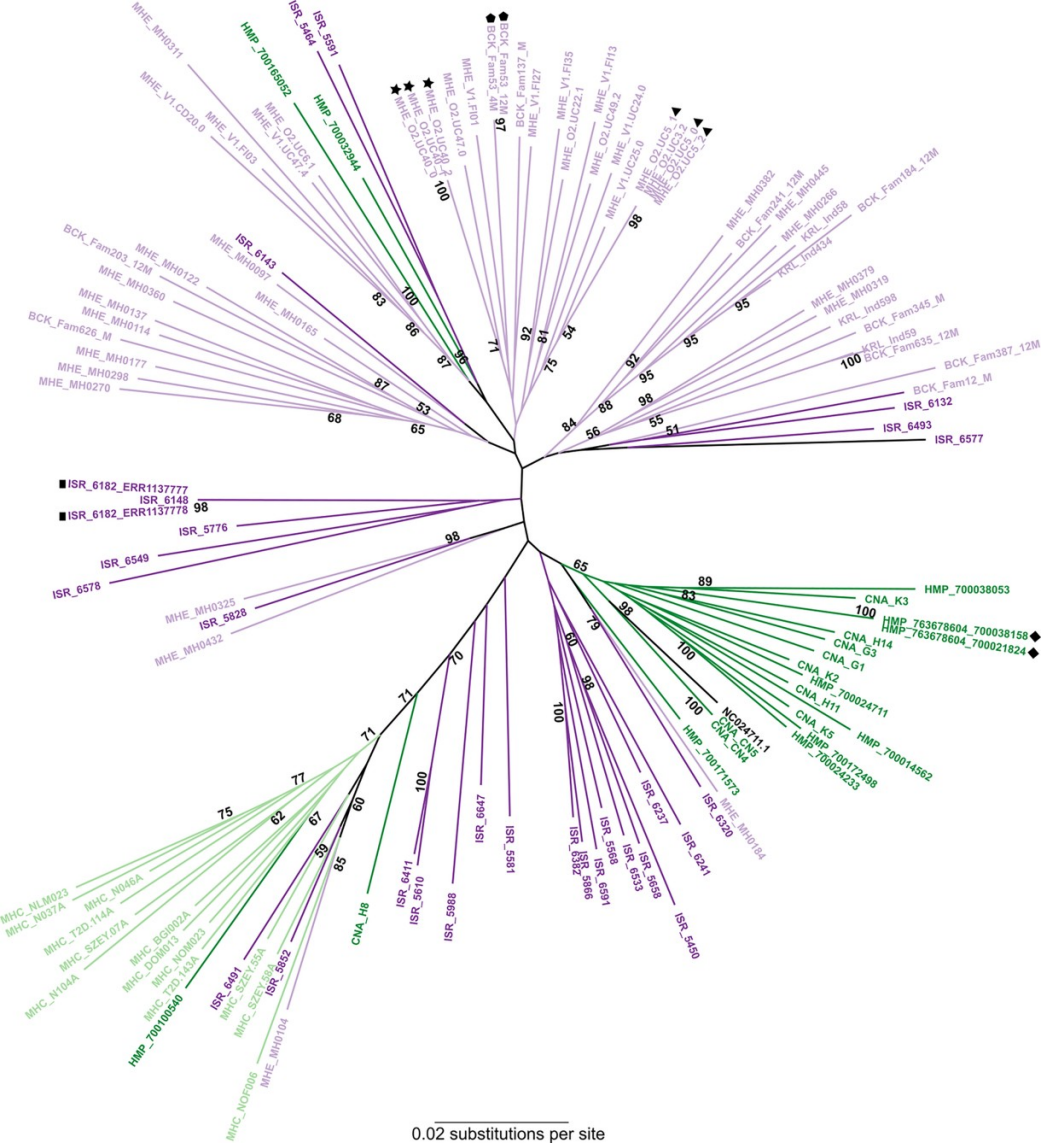
Phylogenomic analysis of crAssphage strains. The Maximum Likelihood tree was based on a multi-genome alignment comprising 97,065 sites. The tree was built using the Generalized Time-Reversible model with gamma-distributed rate variation and proportion of invariant sites. Bootstrap support was estimated from 100 replicates; only values greater than 50% are shown. Strains are color-coded according to the geographic location: dark green–The Americas, light green–Asia, light violet–Europe, and dark purple–Middle East (Israel). Symbols are used to denote strains from the same individual.

To assess whether the lack of biogeographic structure in the multi-gene phylogeny was due to lack of sufficient information for phylogenetic analysis, we identified a subset of 118 samples fulfilling the following criteria: 1) according to the *de novo* assembly results, none of crAssphage proteins were matched by more than one sample ORF, and 2) according to the reference-based mapping, more than 70% of the crAssphage genome was covered at least 10-fold. Furthermore, we determined that these strains showed a range of 2–280 heterozygous sites (i.e. a maximum of 0.3% of sites across the genome were heterozygous). Taken altogether, we considered these samples to contain only one dominant crAssphage strain. The reference crAssphage genome was also included. A multi-genome alignment comprising all 97,065 nucleotide sites was built and used to generate an ML tree (Fig 1). This phylogeny supports the findings of our multi-gene analysis. CrAssphage strains from the same individual cluster together, with high bootstrap support. Strains from individuals from the same study dataset do not all necessarily cluster together; many such phylogenetically dissimilar strains are found in individuals within a study population. At the level of broadly defined geographic regions, some biogeographic structure is observed. However, even at phylogenomic resolution, due to low bootstrap values, the relative positions of these clusters within the crAssphage phylogeny cannot be well-defined.

### CrAssphage population expansion

We hypothesized that the lack of strong biogeographic clustering could be explained by a recent expansion event in crAssphage. To asses this, we used a Bayesian Evolutionary Analysis Sampling Trees (BEAST) approach [46]. The analysis was performed using the multi-gene alignment described previously. Because a substitution rate for crAssphage *sensu stricto* has not yet been determined, we used a substitution rate recently estimated for the crAss-like family of bacteriophages [47]. Our Bayesian Skyline plot (S3 Fig) shows that crAssphage strains underwent a recent expansion event, sometime within the past hundred years. We stress the uncertainty of this estimate given the limitations of our Bayesian dating approach–namely, the lack of appropriate external calibration points and a substitution rate specific to crAssphage *sensu stricto*. However, we note that our analysis shows that this population expansion occurred recently, i.e. within the past few hundred years, consistent with the advance of industrialization, as opposed to having occurred thousands of years ago. Given the recent successful propagation of crAss001, a member of the crAss-like family of phages, in culture [13], the culturing of crAssphage and determination of its substitution rate has become a possibility. Further investigation using a crAssphage-specific substitution rate will provide a more robust estimate of the timing of the population expansion.

### CrAssphage acquisition and persistence

Acquisition and persistence of crAssphage strains in the human gut was evaluated using data from studies focusing on 1) mother-infant pairs, 2) twin-pairs, and 3) longitudinal sampling of individuals. We assessed the potential to identify vertical transmission of crAssphage using the BCK dataset [22], which comprises samples from healthy Swedish mothers (N = 100) and their infants (N = 100) at birth, 4-months, and 12-months of age. CrAssphage prevalence among the healthy mothers was 23%. None of the samples from newborns were considered crAss-positive. Most of the samples from newborn infants showed no reads mapping to crAssphage and no crAssphage proteins recovered from the *de novo* metagenomic assembly approach (S2 Table), with the exception of the infants from Family 549 and Family 263, who showed presence of three and five crAssphage proteins, respectively, at birth. The mothers from both families were also crAss-positive. The infant from Family 263 was determined to be crAss-positive at the four- and 12-month stages, whereas the infant from Family 549 was not considered crAss-positive according to our criteria. At the four-month stage, a total of three infants were crAss-positive, all of whom remained crAss-positive at the 12-month stage as well. A total of 16 infants were crAss-positive at the 12-month stage, suggesting that by the end of the first year of life, crAssphage prevalence among infants was similar to that in mothers (chi-square = 1.5608; p-value = 0.2115, α = 0.05). Interestingly, in nine out of 16 mother-infant pairs, the 12-month old infants were crAss-positive whereas mothers were completely crAss-negative (no crAssphage proteins were recovered from the samples from the mothers). This supports the hypothesis that crAssphage may be acquired by means other than vertical transmission, as recently reported by [21].

**Fig 2:**
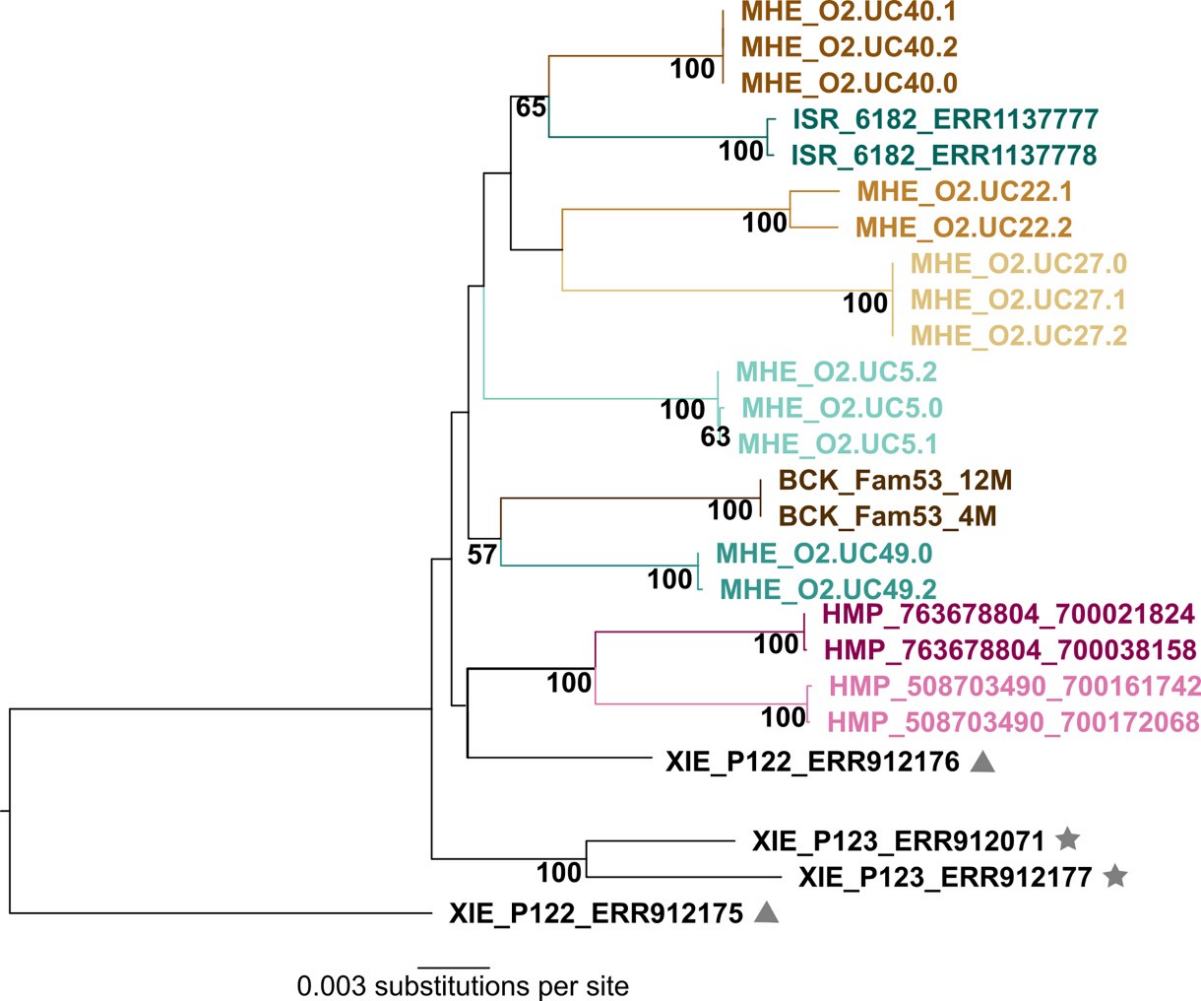
Relationships of crAssphage strains recovered from the same individual and twin-pairs. The Maximum Likelihood tree was based on the multi-gene alignment comprising 12,642 nucleotide sites. Sites with missing data were eliminated. The tree was built using the Generalized Time-Reversible model with gamma-distributed rate variation and proportion of invariant sites. Bootstrap support values estimated from 100 replicates are given; only values greater than 50% are shown. CrAssphage strains recovered from multiple samples from the same individual are color-coded accordingly (MHE, ISR, HMP, and BCK datasets). Symbols are used to denote crAssphage strains recovered from the same twin-pair (XIE dataset).

To study concordance of crAssphage prevalence among twin-pairs, we screened the XIE dataset comprising samples from twin-pairs from the UK (N = 124 pairs). We found 15 cases wherein both twins were crAss-positive and 57 cases wherein both twins were crAss-negative. There were 51 twin-pairs with discordant crAss-positive status. CrAssphage strains from only two crAss-positive twin-pairs could be included in our phylogenetic analyses: in one case, the strains from twin-pair P98 clustered together, whereas those from twin-pair P122 did not (Fig 2).

Additionally, we looked for signatures of crAssphage strain continuity among individuals using the BCK, HMP, and MHE datasets. As seen in Fig 2, crAssphage strains recovered at different time-points from the same individual cluster together, as also reported by [16]. In certain individuals (for example, MHE_O2.UC40), crAssphage strains from different time-points are a 100% identical, suggesting persistence of a single crAssphage strain. In others (for example, MHE_O2.UC22), the strains are closely related but not identical, suggesting that at different time-points, different crAssphage strains might be dominant in an individual. Similar findings have been reported for crAss-like phages by a recent longitudinal study of gut virome diversity [47].

## Conclusions

The geographic distribution of crAssphage is global [14, 16], but as observed here, the prevalence of crAssphage is lower within samples from more traditional, hunter-gatherer populations such as the Hadza from Tanzania and Matses from Peru. The overall picture from the data presented here is that crAssphage prevalence is associated with an industrialized lifestyle/diet, but with no associations to health, age, sex, or body-size variables. CrAssphage strains from the same individual tend to cluster together phylogenetically. Overall, crAssphage shows limited biogeographic clustering as seen in cases of a recent population expansion event. We estimate that this expansion occurred approximately within the past few hundred years; however, the mechanism behind this expansion remains uncertain. Alternatively, this pattern can be attributed to a passive effect of a population expansion within its bacterial host(s) rather than an active expansion event within the bacteriophage. Future research elucidating what bacterial species serve as hosts of crAssphage *sensu stricto* will open avenues for exploring such hypotheses regarding host-bacteriophage co-evolution.

## Methods

### Data acquisition and processing

Gut metagenomic data for a total of 3,341 samples from were downloaded from the Sequence Read Archive or European Nucleotide Archive (Table 1). Shotgun data were processed using AdapterRemoval v2 [48] to remove reads with ambiguous bases (‘N”), trim at low-quality bases (Q<30), and merge overlapping read-pairs. Processed reads longer than 30 base pairs (bp) were retained for downstream analysis. These “analysis-ready” reads were then screened for the presence of crAssphage.

### Reference-based mapping

The analysis-ready reads were mapped to the reference crAssphage genome (NC024711.1) using Bowtie2 [37] with the “no-unal” option to discard unmapped reads. The resulting SAM files were processed using SAMTools v1.3 [49], converted into BAM files, quality-filtered at Phred threshold 37, and duplicate reads were removed using rmdup. SAMTools mpileup and VarScan v2.4.3 [50] were used to generate a VCF file with the following parameters: minimum coverage: 10, minimum coverage of variant allele: 3; minimum average quality: 30, minimum variant allele frequency: 0.2, minimum frequency for homozygotes: 0.9, p-value: 1, and strand filter: 0. This VCF file comprised both variant and invariant sites present in the reference crAssphage genome, resulting in a total of 97,065 sites. A custom perl script was used to generate a FASTA file containing the complete genome of the crAssphage strain from the VCF file. The number of heterozygous sites was used to assess presence of one or multiple crAssphage strains in the sample.

### *De novo* metagenomic assembly

For each sample, the analysis-ready reads were independently assembled into contigs using MEGAHIT [38]. Depth of coverage was calculated by mapping analysis-ready reads to assembled contigs using Bowtie2 [37], followed by processing of resulting alignment files using SAMTools [49] and custom R scripts. Open reading frame (ORF) prediction was carried out using Prodigal [51]. A custom BLAST database was created using the collection of 90 proteins previously predicted from the crAssphage reference genome (NC_024711.1) [12]. The predicted ORFs (amino acid) from each shotgun metagenome were queried against this custom database using BLASTP [39], and matches were identified using the following criteria: 1) query coverage (length of alignment / query length) ≥ 95%, 2) percent identity ≥ 95%, and 3) E value < 1e-5. A sample was considered crAss-positive if matches were recovered for at least 33 reference crAssphage proteins.

### Association of crAssphage prevalence with metadata variables

Individuals were grouped on the basis of 1) health status, 2) age: < 18 years, 18–40 years, 41–65 years, and > 65 years, 3) sex: male and female, and 4) BMI: underweight (BMI < 18.5), normal (BMI 18.5–24.99), and overweight (BMI ≥ 25), according to the World Health Organization recommendations. Associations between the prevalence of crAssphage and health status, age, sex, and BMI categories were assessed using the Chi square test in R [52].

### Gut bacterial taxonomic profiles

For the MAT dataset [31], analysis-ready reads for each individual were mapped to the Greengenes database of 16S rRNA gene sequences [53] using Bowtie2 [37]. Unmapped reads were removed using the—no-unal option. The resulting SAM files were converted to BAM files, sorted, and duplicates were removed using SAMTools v1.3 [49]. The sequences of the reads mapping to the Greengenes database were obtained from the BAM files. These sequences were clustered into Operational Taxonomic Units (OTUs) using vsearch [54], employing the cluster_fast algorithm and comparing to the GreenGenes database. Other parameters used included a minimum sequence length of 70 bp and 97% similarity for clustering. The resulting OTU table was rarefied to a depth of 10,000 sequences per sample and singleton OTUs were removed using QIIME [55]. Taxonomic summaries were generated at the phylum- and genus-levels. Relative abundances of genus *Bacteroides* in individuals were determined and plotted using R [52].

### Multi-gene phylogenetic analysis

For each assembled metagenome, the number of unique ORFs matching each crAssphage reference protein was calculated from the BLASTP results (S2 Table). Only samples identified as containing one unique ORF matching each crAssphage reference protein were selected for phylogenetic analysis. Depth of coverage information was used to verify that identified ORFs were representative of the same viral genome.

A previously published study demonstrated the use of putative capsid proteins (genes 75, 76, 77, 78, and 79) to document diversity among crAss-like phages [12]. Multi-gene phylogenetic analyses were conducted using these capsid protein-encoding genes, as well as five other genes found in members of the crAss-like phage family. These included genes encoding hypothetical proteins (gene 20 and gene 23), single-stranded DNA-binding protein (gene 21), DNA-G family primase (gene 22), and PD-(D/E)XK family nuclease (gene 85). A subset of 232 samples and the reference crAssphage genome was included in this analysis. The gene sequences were aligned separately using MUSCLE v.3.8.31 with default parameters [56] and concatenated together. Sites with missing data and gaps were completely removed. A Maximum Likelihood (ML) tree was built using RAXML v8.2.4 [57], using the Generalized Time-Reversible model with gamma-distributed rate variation and proportion of invariable sites (GTR+G+I). Bootstrap support was estimated from 100 replicates.

### Genome-based phylogenetic analysis

A subset of 118 samples determined to carry only one crAssphage strain was selected. A whole-genome alignment of all strains was used as input for RAXML [57], and a ML tree was generated using the GTR+G+I model and 100 bootstrap replicates.

### Timing of crAssphage population expansion

To estimate the timing of a potential population expansion among crAssphage sequences, the multi-gene alignment was used as input for BEAST v1.8.4 [46]. We used a mean substitution rate of 6 x 10^−4^ substitutions per site per year, with a standard deviation of 5.1 x 10^−4^, as estimated for the crAss-like family of bacteriophages [47]. We used a GTR+G+I model of nucleotide substitution, an uncorrelated lognormal clock model with uniform rate across branches, and a Bayesian Coalescent Skyline plot tree prior. One Markov Chain Monte Carlo (MCMC) run was carried out with 100,000,000 iterations, sampling every 10,000 steps. The first 10,000,000 iterations were discarded as burn-in. Tracer [58] was used to visualize the results of the MCMC run and generate a Bayesian Skyline Plot.

## Supporting information

S1 Table S1 Table contains a list of all 3,341 samples included in this study, associated meta-data, and results of the crAssphage screening using reference-based mapping and *de novo* metagenomic assembly. (XLSX)

S2 Table S2 Table shows the hits for all crAssphage reference proteins across the samples. (XLSX)

S1 Fig Relative abundance of genus *Bacteroides* among Matses individuals. Boxplot denoting percentage relative abundance of *Bacteroides* sp. among Matses individuals. Values corresponding to crAss-positive individuals are denoted in red.
(PDF)

S2 Fig Multi-gene phylogenetic analysis of 233 crAssphage strains. The Maximum Likelihood tree was based on the multi-gene alignment comprising 12,642 nucleotide sites. Sites with missing data were eliminated. The tree was built using the Generalized Time-Reversible model with gamma-distributed rate variation and proportion of invariant sites. Bootstrap support values estimated from 100 replicates are given; only values greater than 50% are shown. Strains are color-coded according to the geographic location: dark green–The Americas, light green–Asia, light violet–Europe, and dark purple–Middle East (Israel). The differentially-colored symbols next to the taxa names are used to denote strains from the same individual.
(PDF)

S3 Fig Bayesian Skyline Plot of crAssphage strains. The X-axis denotes time in years before present (YBP) and Y-axis denotes estimated effective population size. The blue shaded region denotes the 95% Highest Posterior Density interval.
(PDF)

## References

[1] Virgin HW. The virome in mammalian physiology and disease. Cell. 2014;157(1):142–50. 10.1016/j.cell.2014.02.032 24679532 PMC3977141

[2] Rohwer F, Youle M. Consider something viral in your research. Nat Rev Micro. 2011;9(5):308–9. 10.1038/nrmicro2563 21612126

[3] De Paepe M, Leclerc M, Tinsley CR, Petit MA. Bacteriophages: an underestimated role in human and animal health? Frontiers in cellular and infection microbiology. 2014;4:39 10.3389/fcimb.2014.00039 24734220 PMC3975094

[4] Reyes A, Semenkovich NP, Whiteson K, Rohwer F, Gordon JI. Going viral: next-generation sequencing applied to phage populations in the human gut. Nat Rev Microbiol. 2012;10(9):607–17. 10.1038/nrmicro2853 22864264 PMC3596094

[5] Williams SC. The other microbiome. Proc Natl Acad Sci U S A. 2013;110(8):2682–4. 10.1073/pnas.1300923110 23388628 PMC3581985

[6] Modi SR, Lee HH, Spina CS, Collins JJ. Antibiotic treatment expands the resistance reservoir and ecological network of the phage metagenome. Nature. 2013;499(7457):219 10.1038/nature12212 23748443 PMC3710538

[7] Manrique P, Bolduc B, Walk ST, van der Oost J, de Vos WM, Young MJ. Healthy human gut phageome. Proceedings of the National Academy of Sciences. 2016;113(37):10400. 10.1073/pnas.1601060113 PMC5027468 27573828

[8] Shkoporov AN, Hill C. Bacteriophages of the Human Gut: The "Known Unknown" of the Microbiome. Cell Host Microbe. 2019;25(2):195–209. 10.1016/j.chom.2019.01.017 30763534

[9] Dutilh BE, Cassman N, McNair K, Sanchez SE, Silva GGZ, Boling L, et al A highly abundant bacteriophage discovered in the unknown sequences of human faecal metagenomes. Nat Commun. 2014;5. 10.1038/ncomms5498 PMC4111155 25058116

[10] Reyes A, Haynes M, Hanson N, Angly FE, Heath AC, Rohwer F, et al Viruses in the faecal microbiota of monozygotic twins and their mothers. Nature. 2010;466(7304):334–8. 10.1038/nature09199 20631792 PMC2919852

[11] Group NHW, Peterson J, Garges S, Giovanni M, McInnes P, Wang L, et al The NIH Human Microbiome Project. Genome Res. 2009;19(12):2317–23. 10.1101/gr.096651.109 19819907 PMC2792171

[12] Yutin N, Makarova KS, Gussow AB, Krupovic M, Segall A, Edwards RA, et al Discovery of an expansive bacteriophage family that includes the most abundant viruses from the human gut. Nat Microbiol. 2018;3(1):38–46. 10.1038/s41564-017-0053-y 29133882 PMC5736458

[13] Shkoporov AN, Khokhlova EV, Fitzgerald CB, Stockdale SR, Draper LA, Ross RP, et al ΦCrAss001 represents the most abundant bacteriophage family in the human gut and infects Bacteroides intestinalis. Nature Communications. 2018;9(1):4781 10.1038/s41467-018-07225-7 30429469 PMC6235969

[14] Cinek O, Mazankova K, Kramna L, Odeh R, Alassaf A, Ibekwe MU, et al Quantitative CrAssphage real-time PCR assay derived from data of multiple geographically distant populations. J Med Virol. 2018;90(4):767–71. 10.1002/jmv.25012 29297933

[15] Holmfeldt K, Solonenko N, Shah M, Corrier K, Riemann L, VerBerkmoes NC, et al Twelve previously unknown phage genera are ubiquitous in global oceans. Proceedings of the National Academy of Sciences. 2013;110(31):12798. 10.1073/pnas.1305956110 PMC3732932 23858439

[16] Edwards RA, Vega AA, Norman HM, Ohaeri M, Levi K, Dinsdale EA, et al Global phylogeography and ancient evolution of the widespread human gut virus crAssphage. Nature Microbiology. 2019. 10.1038/s41564-019-0494-6 PMC7440971 31285584

[17] Ahmed W, Lobos A, Senkbeil J, Peraud J, Gallard J, Harwood VJ. Evaluation of the novel crAssphage marker for sewage pollution tracking in storm drain outfalls in Tampa, Florida. Water Res. 2017;131:142–50. 10.1016/j.watres.2017.12.011 29281808

[18] Garcia-Aljaro C, Balleste E, Muniesa M, Jofre J. Determination of crAssphage in water samples and applicability for tracking human faecal pollution. Microb Biotechnol. 2017;10(6):1775–80. 10.1111/1751-7915.12841 28925595 PMC5658656

[19] Stachler E, Kelty C, Sivaganesan M, Li X, Bibby K, Shanks OC. Quantitative CrAssphage PCR Assays for Human Fecal Pollution Measurement. Environ Sci Technol. 2017;51(16):9146–54. 10.1021/acs.est.7b02703 28700235 PMC7350147

[20] Farkas K, Adriaenssens EM, Walker DI, McDonald JE, Malham SK, Jones DL. Critical Evaluation of CrAssphage as a Molecular Marker for Human-Derived Wastewater Contamination in the Aquatic Environment. Food and Environmental Virology. 2019. 10.1007/s12560-019-09369-1 PMC6513805 30758724

[21] Tamburini FB, Sherlock GJ, Bhatt AS. Transmission and persistence of crAssphage, a ubiquitous human-associated bacteriophage. bioRxiv. 2018:460113.

[22] Backhed F, Roswall J, Peng Y, Feng Q, Jia H, Kovatcheva-Datchary P, et al Dynamics and Stabilization of the Human Gut Microbiome during the First Year of Life. Cell Host Microbe. 2015;17(5):690–703. 10.1016/j.chom.2015.04.004 25974306

[23] Guerin E, Shkoporov A, Stockdale SR, Clooney AG, Ryan FJ, Sutton TDS, et al Biology and Taxonomy of crAss-like Bacteriophages, the Most Abundant Virus in the Human Gut. Cell Host Microbe. 2018;24(5):653–64 e6. 10.1016/j.chom.2018.10.002 30449316

[24] Callahan BJ, McMurdie PJ, Holmes SP. Exact sequence variants should replace operational taxonomic units in marker-gene data analysis. The Isme Journal. 2017;11:2639 10.1038/ismej.2017.119 28731476 PMC5702726

[25] Sankaranarayanan K, Ozga AT, Warinner C, Tito RY, Obregon-Tito AJ, Xu J, et al Gut Microbiome Diversity among Cheyenne and Arapaho Individuals from Western Oklahoma. Curr Biol. 2015;25(24):3161–9. 10.1016/j.cub.2015.10.060 26671671 PMC4703035

[26] Rampelli S, Schnorr Stephanie L, Consolandi C, Turroni S, Severgnini M, Peano C, et al Metagenome Sequencing of the Hadza Hunter-Gatherer Gut Microbiota. Current Biology. 2015;25(13):1682–93. 10.1016/j.cub.2015.04.055 25981789

[27] Smits SA, Leach J, Sonnenburg ED, Gonzalez CG, Lichtman JS, Reid G, et al Seasonal cycling in the gut microbiome of the Hadza hunter-gatherers of Tanzania. Science. 2017;357(6353):802 10.1126/science.aan4834 28839072 PMC5891123

[28] Zeevi D, Korem T, Zmora N, Israeli D, Rothschild D, Weinberger A, et al Personalized Nutrition by Prediction of Glycemic Responses. Cell. 2015;163(5):1079–94. 10.1016/j.cell.2015.11.001 26590418

[29] Karlsson FH, Tremaroli V, Nookaew I, Bergstrom G, Behre CJ, Fagerberg B, et al Gut metagenome in European women with normal, impaired and diabetic glucose control. Nature. 2013;498(7452):99–103. 10.1038/nature12198 23719380

[30] Liu W, Zhang J, Wu C, Cai S, Huang W, Chen J, et al Unique Features of Ethnic Mongolian Gut Microbiome revealed by metagenomic analysis. Sci Rep. 2016;6:34826 10.1038/srep34826 27708392 PMC5052615

[31] Obregon-Tito AJ, Tito RY, Metcalf J, Sankaranarayanan K, Clemente JC, Ursell LK, et al Subsistence strategies in traditional societies distinguish gut microbiomes. Nat Commun. 2015;6:6505 10.1038/ncomms7505 25807110 PMC4386023

[32] Qin J, Li Y, Cai Z, Li S, Zhu J, Zhang F, et al A metagenome-wide association study of gut microbiota in type 2 diabetes. Nature. 2012;490(7418):55–60. 10.1038/nature11450 23023125

[33] Le Chatelier E, Nielsen T, Qin J, Prifti E, Hildebrand F, Falony G, et al Richness of human gut microbiome correlates with metabolic markers. Nature. 2013;500(7464):541–6. 10.1038/nature12506 23985870

[34] Li J, Jia H, Cai X, Zhong H, Feng Q, Sunagawa S, et al An integrated catalog of reference genes in the human gut microbiome. Nat Biotechnol. 2014;32(8):834–41. 10.1038/nbt.2942 24997786

[35] Nielsen HB, Almeida M, Juncker AS, Rasmussen S, Li J, Sunagawa S, et al Identification and assembly of genomes and genetic elements in complex metagenomic samples without using reference genomes. Nat Biotechnol. 2014;32(8):822–8. 10.1038/nbt.2939 24997787

[36] Xie H, Guo R, Zhong H, Feng Q, Lan Z, Qin B, et al Shotgun Metagenomics of 250 Adult Twins Reveals Genetic and Environmental Impacts on the Gut Microbiome. Cell Syst. 2016;3(6):572–84 e3. 10.1016/j.cels.2016.10.004 27818083 PMC6309625

[37] Langmead B, Salzberg SL. Fast gapped-read alignment with Bowtie 2. Nature Methods. 2012;9:357 10.1038/nmeth.1923 22388286 PMC3322381

[38] Li D, Liu C-M, Luo R, Sadakane K, Lam T-W. MEGAHIT: an ultra-fast single-node solution for large and complex metagenomics assembly via succinct de Bruijn graph. Bioinformatics. 2015;31(10):1674–6. 10.1093/bioinformatics/btv033 25609793

[39] Altschul SF, Gish W, Miller W, Myers EW, Lipman DJ. Basic local alignment search tool. Journal of Molecular Biology. 1990;215(3):403–10. 10.1016/S0022-2836(05)80360-2 2231712

[40] Adriaenssens E, Brister JR. How to Name and Classify Your Phage: An Informal Guide. Viruses. 2017;9(4):70. 10.3390/v9040070 PMC5408676 28368359

[41] Schnorr SL, Candela M, Rampelli S, Centanni M, Consolandi C, Basaglia G, et al Gut microbiome of the Hadza hunter-gatherers. Nature Communications. 2014;5:3654 10.1038/ncomms4654 24736369 PMC3996546

[42] Yatsunenko T, Rey FE, Manary MJ, Trehan I, Dominguez-Bello MG, Contreras M, et al Human gut microbiome viewed across age and geography. Nature. 2012;486:222 10.1038/nature11053 22699611 PMC3376388

[43] Maixner F, Krause-Kyora B, Turaev D, Herbig A, Hoopmann MR, Hallows JL, et al The 5300-year-old Helicobacter pylori genome of the Iceman. Science. 2016;351(6269):162 10.1126/science.aad2545 26744403 PMC4775254

[44] Ozga AT. Viral Metagenomics and Anthropology in the Americas [Ph.D. dissertation]. Norman: University of Oklahoma; 2015.

[45] Gupta VK, Paul S, Dutta C. Geography, Ethnicity or Subsistence-Specific Variations in Human Microbiome Composition and Diversity. Front Microbiol. 2017;8:1162 10.3389/fmicb.2017.01162 28690602 PMC5481955

[46] Drummond AJ, Rambaut A. BEAST: Bayesian evolutionary analysis by sampling trees. BMC Evolutionary Biology. 2007;7(1):214. 17996036 10.1186/1471-2148-7-214 PMC2247476

[47] Shkoporov AN, Clooney AG, Sutton TDS, Ryan FJ, Daly KM, Nolan JA, et al The Human Gut Virome Is Highly Diverse, Stable, and Individual Specific. Cell Host & Microbe. 2019;26(4):527–41.e5. 31600503 10.1016/j.chom.2019.09.009

[48] Schubert M, Lindgreen S, Orlando L. AdapterRemoval v2: rapid adapter trimming, identification, and read merging. BMC Research Notes. 2016;9 10.1186/s13104-015-1814-4 26868221 PMC4751634

[49] Li H, Handsaker B, Wysoker A, Fennell T, Ruan J, Homer N, et al The Sequence Alignment/Map format and SAMtools. Bioinformatics. 2009;25(16):2078–9. 10.1093/bioinformatics/btp352 19505943 PMC2723002

[50] Koboldt DC, Zhang Q, Larson DE, Shen D, McLellan MD, Lin L, et al VarScan 2: Somatic mutation and copy number alteration discovery in cancer by exome sequencing. Genome Research. 2012;22(3):568–76. 10.1101/gr.129684.111 22300766 PMC3290792

[51] Hyatt D, Chen G-L, LoCascio PF, Land ML, Larimer FW, Hauser LJ. Prodigal: prokaryotic gene recognition and translation initiation site identification. BMC Bioinformatics. 2010;11(1):119. 20211023 10.1186/1471-2105-11-119 PMC2848648

[52] Team RC. R: A language and environment for statistical computing. Vienna, Austria: R Foundation for Statistical Computing; 2010.

[53] DeSantis TZ, Hugenholtz P, Larsen N, Rojas M, Brodie EL, Keller K, et al Greengenes, a chimera-checked 16S rRNA gene database and workbench compatible with ARB. Appl Environ Microbiol. 2006;72(7):5069–72. 10.1128/AEM.03006-05 16820507 PMC1489311

[54] Rognes T, Flouri T, Nichols B, Quince C, Mahé F. VSEARCH: a versatile open source tool for metagenomics. PeerJ. 2016;4:e2584 10.7717/peerj.2584 27781170 PMC5075697

[55] Caporaso JG, Kuczynski J, Stombaugh J, Bittinger K, Bushman FD, Costello EK, et al QIIME allows analysis of high-throughput community sequencing data. Nature Methods. 2010;7:335 10.1038/nmeth.f.303 20383131 PMC3156573

[56] Edgar RC. MUSCLE: multiple sequence alignment with high accuracy and high throughput. Nucleic Acids Research. 2004;32(5):1792–7. 10.1093/nar/gkh340 15034147 PMC390337

[57] Stamatakis A. RAxML version 8: a tool for phylogenetic analysis and post-analysis of large phylogenies. Bioinformatics. 2014;30(9):1312–3. 10.1093/bioinformatics/btu033 24451623 PMC3998144

[58] Rambaut A, Drummond AJ, Xie D, Baele G, Suchard MA. Posterior Summarization in Bayesian Phylogenetics Using Tracer 1.7. Systematic Biology. 2018:syy032–syy. 10.1093/sysbio/syy032 PMC6101584 29718447

