# Supplementary figures and images for: Biogeographic study of human gut-associated crAssphage suggests impacts from industrialization and recent expansion
Source: PLoS One. 2020 Jan 15;15(1):e0226930. doi: 10.1371/journal.pone.0226930 (PMC6961876; doi:10.1371/journal.pone.0226930)

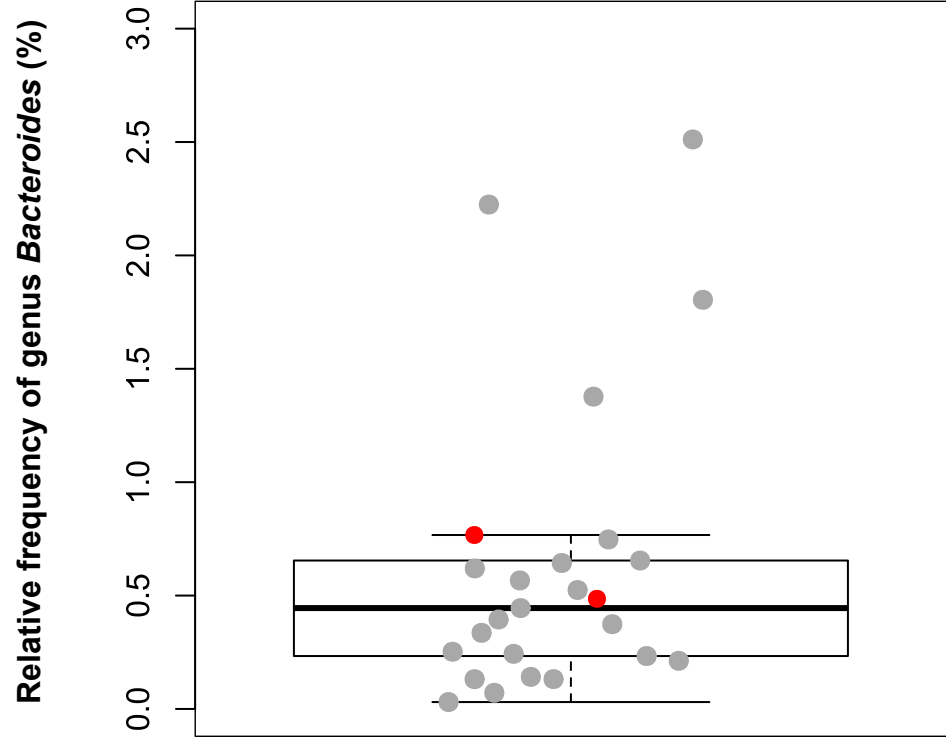

Supplement: S1 Fig — Boxplot denoting percentage relative abundance of Bacteroides sp. among Matses individuals. Values corresponding to crAss-positive individuals are denoted in red. (PDF) [file pone.0226930.s003.pdf]

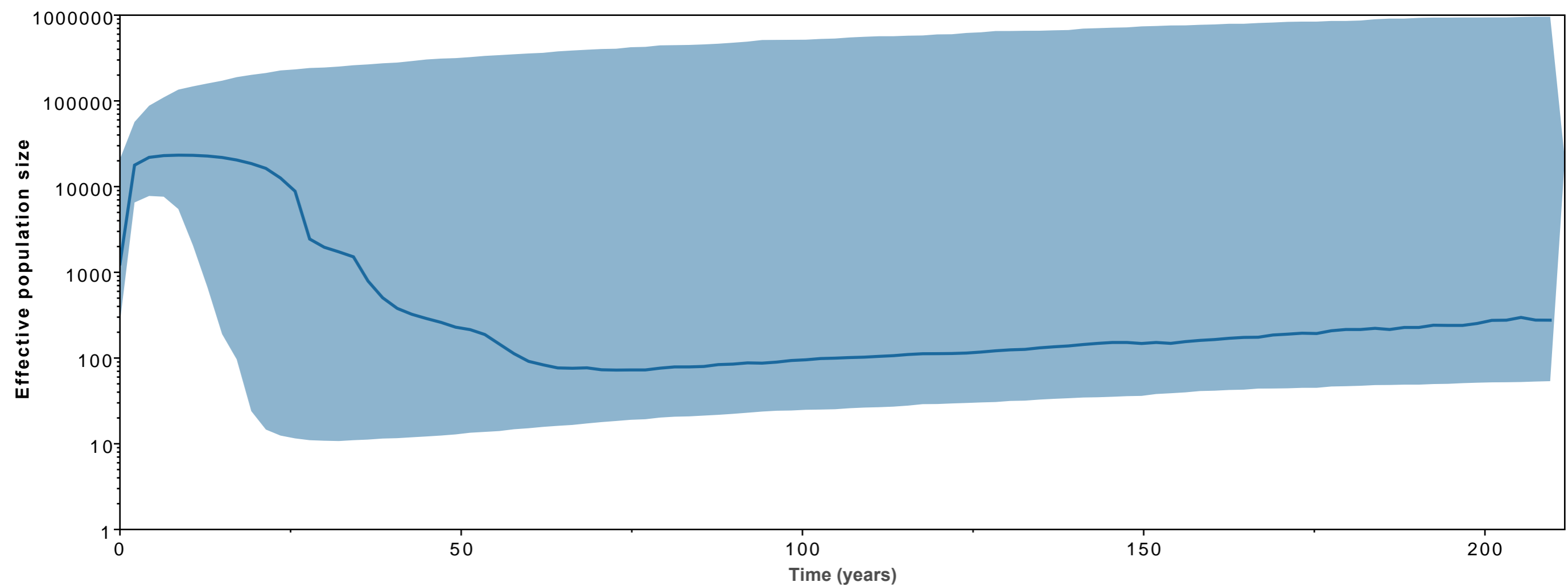

Supplement: S3 Fig — The X-axis denotes time in years before present (YBP) and Y-axis denotes estimated effective population size. The blue shaded region denotes the 95% Highest Posterior Density interval. (PDF) [file pone.0226930.s005.pdf]
