# Supplementary material for: Biogeographic study of human gut-associated crAssphage suggests impacts from industrialization and recent expansion
Source: PLoS One. 2020 Jan 15;15(1):e0226930. doi: 10.1371/journal.pone.0226930 (PMC6961876; doi:10.1371/journal.pone.0226930)
Supplement: S2 Fig — The Maximum Likelihood tree was based on the multi-gene alignment comprising 12,642 nucleotide sites. Sites with missing data were eliminated. The tree was built using the Generalized Time-Reversible model with gamma-distributed rate variation and proportion of invariant sites. Bootstrap support values estimated from 100 replicates are given; only values greater than 50% are shown. Strains are color-coded according to the geographic location: dark green–The Americas, light green–Asia, light violet–Europe, and dark purple–Middle East (Israel). The differentially-colored symbols next to the taxa names are used to denote strains from the same individual. (PDF) [file pone.0226930.s004.pdf]

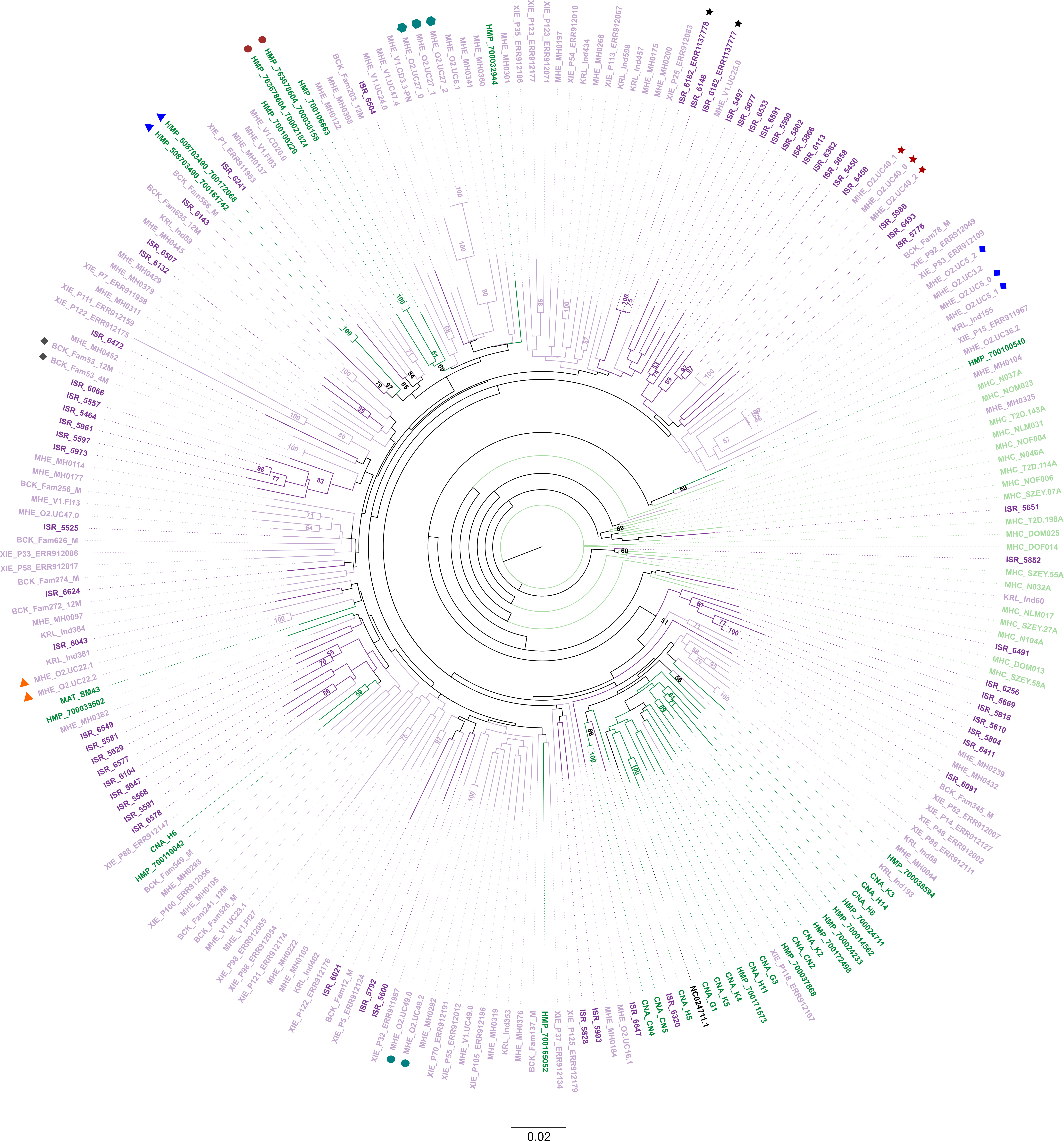

HMP_508703490_700172068
HMP_508703490_700161742
XIE_P1_ERR911953
MHE_MH0137
MHE_V1.FI03
MHE_V1.CD20.0
HMP_700106229
HMP_763678604_700021824
HMP_763678604_700038158
HMP_700106663
MHE_MH0122
BCK_Fam203_12M
MHE_MH0398
ISR_6504
MHE_V1.UC24.0
MHE_V1.UC47.4
MHE_V1.CD3.3-PN
MHE_O2.UC27_0
MHE_O2.UC27_1
MHE_O2.UC27_2
MHE_O2.UC6.1
MHE_MH0341
MHE_MH0360
HMP_700032944
MHE_MH0301
XIE_P35_ERR912186
XIE_P123_ERR912177
XIE_P123_ERR912071
MHE_MH0197
XIE_P54_ERR912010
KRL_Ind434
MHE_MH0266
XIE_P113_ERR912067
KRL_Ind598
KRL_Ind457
MHE_MH0175
MHE_MH0200
XIE_P25_ERR912083
ISR_6182_ERR1137778
ISR_6148
ISR_6182_ERR1137777
MHE_V1.UC25.0
ISR_5497
ISR_5677
ISR_6533
ISR_6591
ISR_5599
ISR_5802
ISR_5866
ISR_6113
ISR_6382
ISR_5658
ISR_5450
ISR_6458
MHE_O2.UC40_1
MHE_O2.UC40_0
MHE_O2.UC40_2
ISR_5988
ISR_6493
ISR_5776
BCK_Fam78_M
XIE_P92_ERR912049
XIE_P83_ERR912109
MHE_O2.UC5_2
MHE_O2.UC3.2
MHE_O2.UC5_0
MHE_O2.UC5_1
KRL_Ind155
XIE_P15_ERR911967
MHE_O2.UC36.2
HMP_700100540
MHE_MH0104
MHC_N037A
MHC_NOM023
MHE_MH0325
MHC_T2D.143A
MHC_NLM031
MHC_NOF004
MHC_N046A
MHC_T2D.114A
MHC_NOF006
MHC_SZEY.07A
ISR_5651
MHC_T2D.198A
MHC_DOM025
MHC_DOF014
ISR_5852
MHC_SZEY.55A
MHC_N032A
KRL_Ind60
MHC_NLM017
MHC_SZEY.27A
MHC_N104A
ISR_6491
MHC_DOM013
MHC_SZEY.58A
ISR_6256
ISR_5669
ISR_5818
ISR_5610
ISR_5804
ISR_6411
MHE_MH0239
MHE_MH0432
ISR_6091
BCK_Fam345_M
XIE_P52_ERR912007
XIE_P14_ERR912127
XIE_P48_ERR912002
XIE_P85_ERR912111
KRL_Ind58
MHE_MH0044
KRL_Ind193
HMP_700038594
CNA_K3
CNA_H14
CNA_H8
HMP_700024711
HMP_700014562
HMP_700024233
HMP_700172498
CNA_K2
CNA_CN2
HMP_700037868
XIE_P118_ERR912167
CNA_G3
CNA_H11
HMP_700171573
CNA_K4
CNA_K5
CNA_G1
NC024711.1
CNA_H5
ISR_6320
CNA_CN5
CNA_CN4
ISR_6647
MHE_O2.UC16.1
MHE_MH0184
ISR_5993
ISR_5828
XIE_P125_ERR912179
XIE_P37_ERR912134
HMP_700165052
BCK_Fam137_M
MHE_MH0376
KRL_Ind353
MHE_MH0319
XIE_P105_ERR912196
MHE_V1.UC49.0
XIE_P55_ERR912012
XIE_P70_ERR912191
MHE_MH0292
MHE_O2.UC49.2
MHE_O2.UC49.0
XIE_P32_ERR911987
ISR_5600
ISR_5792
XIE_P5_ERR912124
BCK_Fam12_M
ISR_6021
XIE_P122_ERR912176
KRL_Ind462
MHE_MH0165
MHE_MH0222
XIE_P121_ERR912174
XIE_P98_ERR912054
XIE_P98_ERR912055
MHE_V1.FI27
MHE_V1.UC23.1
BCK_Fam526_M
BCK_Fam241_12M
MHE_MH0105
XIE_P100_ERR912056
MHE_MH0298
BCK_Fam549_M
HMP_700119042
CNA_H6
XIE_P88_ERR912147
ISR_6578
ISR_5591
ISR_5568
ISR_5647
ISR_6104
ISR_6577
ISR_5629
ISR_5581
ISR_6549
MHE_MH0382
HMP_700033502
MAT_SM43
MHE_O2.UC22.2
MHE_O2.UC22.1
KRL_Ind381
ISR_6043
KRL_Ind384
MHE_MH0097
BCK_Fam272_12M
ISR_6624
BCK_Fam274_M
XIE_P58_ERR912017
XIE_P33_ERR912086
BCK_Fam626_M
ISR_5525
MHE_O2.UC47.0
MHE_V1.FI13
BCK_Fam256_M
MHE_MH0177
MHE_MH0114
ISR_5973
ISR_5597
ISR_5961
ISR_5464
ISR_5557
ISR_6066
BCK_Fam53_4M
BCK_Fam53_12M
MHE_MH0452
ISR_6472
XIE_P122_ERR912175
XIE_P111_ERR912159
MHE_MH0311
XIE_P7_ERR911958
MHE_MH0379
MHE_MH0429
ISR_6132
ISR_6507
MHE_MH0445
KRL_Ind59
BCK_Fam635_12M
ISR_6143
BCK_Fam566_M
ISR_6241
0.02
